# Genetic and Pathogenic Characteristics of an Emerging Lineage 7 PRRSV‐2 (Prime Pac‐Like) Strain in China

**DOI:** 10.1155/tbed/8104154

**Published:** 2025-12-18

**Authors:** Yuan Yang, Haoyu Chen, Feimin Xie, Xuejing Li, Xin Lan, Hua Wang, Ziyi Wang, Kewei Fan, Wei Wei, Cheng Luo, Ailing Dai, Xiaohua Li, Manlin Luo, Chunhua Wei, Jiankui Liu

**Affiliations:** ^1^ College of Life Sciences, Longyan University, Longyan, 364012, China, lyun.edu.cn; ^2^ Fujian Provincial Key Laboratory for the Prevention and Control of Animal Infectious Diseases and Biotechnology, Longyan University, Longyan, 364012, China, lyun.edu.cn; ^3^ College of Animal Science, Fujian Agriculture and Forestry University, Fuzhou, 350002, China, fjau.edu.cn; ^4^ College of Veterinary Medicine, South China Agricultural University, Guangzhou, 510642, China, scau.edu.cn

**Keywords:** NADC30-like, pathogenicity, porcine reproductive and respiratory syndrome virus (PRRSV), Prime Pac-like, recombination

## Abstract

The emergence of the NADC30‐like (Lineage1.8) porcine reproductive and respiratory syndrome virus (PRRSV) has accelerated the evolutionary divergence of PRRSV, causing significant economic losses to Chinese swine industry. In the present study, a novel Lineage 7 (L7/Prime Pac‐like) PRRSV strain FJZHK‐2025 was isolated in China. Full‐length genome analysis revealed that FJZHK‐2025 is closely related to L7 PRRSV and exhibits the highest nucleotide homology (96.4%–96.5%) with the L7 PRRSV reference strains. Notably, FJZHK‐2025 contained a 36 amino acid (aa) insertion within Nsp2 that was identical to the Prime Pac PRRS vaccine strain. Phylogenetic analysis, recombination detection, and regional genomic homology comparisons revealed that FJZHK‐2025 originated from inter‐lineage recombination between L7 and lineage 1.8 (NADC30‐like) PRRSV‐2 strains. Animal challenge experiments showed that piglets infected with FJZHK‐2025 developed a persistent high fever, displayed mild to moderate clinical symptoms and presented with moderate lung pathological lesions, indicating that the FJZHK‐2025 strain is pathogenic to piglets. Therefore, it is critical to develop an effective strategy to prevent and control the spread of L7 PRRSV in China.

## 1. Introduction

Porcine reproductive and respiratory syndrome (PRRS), caused by the PRRS virus (PRRSV), primarily induces reproductive failure (e.g., abortions, early farrowing, weak piglets, and stillbirths) in sows and respiratory symptoms in any age of pig [1–3]. Since the initial reports of PRRS in the USA in 1987 [4] and Europe in the 1990s [5], the disease has spread worldwide, causing huge economic losses to the pig industry [6–8]. According to the latest viral taxonomy, PRRSV can be classified into two distinct species: *Betaarterivirus suid* 1 (PRRSV‐1) and *Betaarterivirus suid* 2 (PRRSV‐2) [9]. PRRSV is a positive‐sense RNA virus with a genome size of ~15.0 kb containing 5′‐untranslated region (UTR), at least 11 known overlapping open reading frames (ORFs) and 3′‐end poly(A) tail [6, 9, 10]. Of these ORFs, ORF1a, and ORF1b encode polyproteins (pp1a and pp1ab) that are cleaved into 16 nonstructural proteins (Nsp1α/1β, Nsp2, Nsp2TF, and Nsp3 to 6, Nsp7α/7β, and Nsp8 to 12). ORF2 to ORF7 encode eight structural proteins of the virus (GP2a, small envelope (E), GP3‐GP5, ORF5a, matrix (M), and nucleocapsid (N) protein) [6, 10].

PRRSV‐2 genome exhibits high genetic variation and can be classified into 11 genetic lineages (L1–L11) [8]. Since it was first reported in 1995, PRRS has been a persistent problem in the Chinese pig industry [11, 12]. The PRRSV strains circulating in China can be divided into four lineages: lineage 8.7 (L8.7/JXA1‐like and CH1a‐like strains), lineage 5.1 (L5.1/VR2332‐like), lineage 3 (L3/QYYZ‐like), and lineage 1 (L1.8/NADC30‐like strains and L1.5/NADC34‐like strains) [12, 13]. NADC30‐like strains (L1.8, first appeared in China in 2012) and NADC34‐like strains (L1.5, emerged in 2017) originating from North America have become the predominant endemic strains, resulting in significant economic losses to Chinese pig farms [12, 14–16]. QYYZ‐like strains (L3) have been prevalent mainly in southern China since they were first reported in 2010 [17]. A highly pathogenic PRRSV strain (HP‐PRRSV/JXA1‐like/L8.7) emerged in China in 2006, leading to a massively destructive outbreak with high mortality in pigs [18]. In addition, Lineage 5 (VR‐2332‐like) strains have remained relatively lower prevalence and limited genetic diversity and have also not caused an epidemic in swine herds since 1996 [12, 19]. Notably, the Prime Pac PRRS vaccine strain and Prime Pac‐like virus, which belongs to lineage 7 (L7) PRRSV‐2, have been used to control PRRSV in the USA [8], but the Prime Pac vaccine is not available in China.

Although Lineage 7 PRRSV was first reported in China in 2008 [20], no further isolates of this virus have been documented since then. Recently, novel recombinant lineage 7 viruses have emerged in China during 2025, causing abortions and stillbirth in pregnant sows, as well as high fever and respiratory disorders in piglets. This novel recombinant PRRSV will make PRRS prevention and control more complex and difficult. Recombination is an important mechanism in the evolution of PRRSV, influencing virus replication, virulence, immune evasion and diagnostic failure [12]. In this study, we isolated and identified a novel L7 PRRSV‐2 strain derived from an inter‐lineage recombinant of the Prime Pac‐like strain (L7) and the Chinese NADC30‐like strain (L1.8), which has not previously been reported in China. Although the detection rate of L7 PRRSV remains low in Chinese pig farms, the possibility of an outbreak of this type of recombinant virus cannot be dismissed. Therefore, in‐depth analysis of the origin, epidemic trend, genetic characteristics and pathogenic properties of this emerging recombinant strain is crucial for updating diagnostic assays and preventing and controlling L7 PRRSV‐2.

## 2. Materials and Methods

### 2.1. Sample Collection

During the routine investigation of L7 PRRSV‐2 epidemiology, a total of 300 samples (lungs, lymph nodes and serum samples) were collected from pig farms in Fujian, Jiangxi, and Guangdong provinces of China from January to May in 2025. The L7 PRRSV‐2 was detected using a PRRSV‐2 quantitative RT‐PCR (RT‐qPCR) assay as previously described [21].

### 2.2. Virus Isolation

In February 2025, tissue samples (e.g., lung tissue, lymphoid tissue, and spleen) and serum were collected from a pig farm exhibiting clinical respiratory signs of PRRS in Fujian Province, China. PRRSV‐2 infection was confirmed by testing samples from affected pigs using a RT‐qPCR assay kit (Qingdao Lijian Bio‐Tech Co. Ltd.) and L7 PRRSV‐2 RT‐qPCR [21]. Additionally, classical swine fever virus (CSFV), porcine circovirus type 2 (PCV2), porcine circovirus type 3 (PCV3), pseudorabies virus (PRV) and African swine fever virus (ASFV) were not detected in the tissue samples. Then, suspensions of lung tissue and serum were passed through a 0.22‐μm filter and inoculated onto MARC‐145 cells and primary porcine alveolar macrophages (PAMs) for virus isolation as described previously [22]. The isolated PRRSV‐2 was confirmed by immunofluorescence assay (IFA) at 72 h postinfection (hpi) [13], and the isolate was designated FJZHK‐2025. Growth properties of FJZHK‐2025 and FJZ03 (a moderately virulent NADC30‐like PRRSV strain isolated in 2013 [16] and used as a positive control) were assessed in PAMs and MARC‐145 cells as previously described [23]. The virus was then purified by plaque assay for next study.

### 2.3. RT‐PCR

Total RNA was extracted from the virus and used for reverse transcription as described previously [13]. The complete genomic sequence of PRRSV was amplified by RT‐PCR with seven pairs of primers designed in our laboratory and re‐sequencing the full genome of PRRSV according to Kvisgaard et al. [24]. The PCR product was purified, cloned and sequenced as described previously [13].

### 2.4. Genomic Sequence Analysis

The complete genome sequence of FJZHK‐2025 was compared with 108 reference PRRSV strains available in GenBank, including 107 different lineages of PRRSV‐2 and one PRRSV‐1. Homology analysis of the nucleotide or aa sequences of each genomic fragment was performed using the MegAlign program in the DNASTAR package. Phylogenetic trees of complete genome and ORF5 were generated using MEGA 7.0 software by the neighbor‐joining (NJ) method. Bootstrap analysis with 1000 replicates was applied to evaluate the robustness of the phylogenetic trees [25].

### 2.5. Recombination Analysis

Recombination events were analyzed using seven recombination detection algorithms (RDP, GENECONV, Bootscan, MaxChi, SiScan, Chimera, and 3Seq) in the RDP4.10 software [26]. Within RDP 4.10, a recombination event was considered authentic if at least five different algorithms detected signals with. Recombination patterns were analyzed using 10 representative strains selected from subtypes L1 to L9 as reference parental strains, including NADC30 (L1.8), NADC34 (L1.5), XW008 (L2), MD001 (L3), EDRD‐1 (L4), VR‐2332 (L5), P129 (L6), Prim Pac (L7), JXA1 (L8), and MN30100 (L9) [27]. PRRSV strains not involved in recombination were then deleted to confirm the final recombination sites. Following this analysis, similarity plot analysis of the full‐length genome sequence of the isolated strain was then performed using Simplot software v3.5.1 to further identify the recombination events and the breakpoint positions [28]. To identify the most closely related parental PRRSV strains, the sequence of the FJZHK‐2025 recombinant fragment was analyzed using the BLAST tool in the GenBank database (http://blast.ncbi.nlm.nih.gov/). The final recombination events were determined using the results from SimPlot 3.5.1, RDP 4.10 and BLAST.

### 2.6. Pathogenicity Study

Nine 4‐week‐old piglets that were negative for PRRSV and PCV2 were divided into two groups: the FJZHK‐2025‐inoculation group (*n* = 5), and the control group (*n* = 4). The FJZHK‐2025‐inoculation group was inoculated intramuscularly and intranasally with FJZHK‐2025‐F3 (the third passage in PAMs cells, 2 × 10^5^ TCID50). The control group was inoculated with an equal volume of RPMI 1640. The piglet in each group was monitored daily for clinical signs, including body temperature, respiratory signs (respiratory condition, cough, and rhinorrhoea), and other general behavior as previously described [29].

Blood samples of each group were collected at 0, 4, 7, 11, 14, 17, and 21 days postinoculation (dpi) for detection of PRRSV‐specific antibody levels by ELISA Kit (IDEXX, USA) and for detection of viral load by RT‐qPCR as previously described [30]. Meanwhile, the weight of all the piglets in each group in the present study was recorded at 0 and 21, and the average daily weight gain (ADWG) was calculated.

Animal trials and sampling procedures in this study were approved by the guidelines of animal ethics committee of Longyan University (Approval Number: LY2025016X).

### 2.7. Pathological Examination

All piglets were euthanized at 21 dpi as previously described [31]. Gross lung lesions were evaluated based on the percentage of affected lung tissue according to a scoring system (score of 0–100) described by Halbur et al. [32]. The lung tissue of the piglets in each group was collected at necropsy. The tissue was then fixed in 10% buffered formalin and processed using standard histopathological protocols. Subsequently, lung tissue sections were subjected to hematoxylin and eosin (HE) staining, and the pathological changes were scored from 0 to 4 as previously described [22]. The PRRSV antigen distribution within the lung samples was detected using immunohistochemistry (IHC) with a polyclonal antibody for the PRRSV N protein (Bioss, Beijing, and China) as previously described [13].

### 2.8. Statistical Analyses

All data in the present study were presented as the mean ± standard deviation (SD). Statistical differences between groups were assessed by one‐way analysis of variance (ANOVA) using GraphPad Prism software (Version 6.0). A *p*‐value <0.05 was considered statistically significant.

## 3. Results

### 3.1. L7 PRRSV‐2 Was Detected in China

During the investigation of L7 PRRSV‐2 epidemiology in 2025, 300 samples were collected from swine farms, with only six samples (2%) were positive.

### 3.2. Virus Isolation

Samples (lung tissue, lymphoid tissue, spleen, and serum) were collected from the PRRS‐affected pig farm, and PRRSV‐2 infection was identified by RT‐qPCR with a CT value of 17.4. PRRSV‐positive sample homogenates were then added to PAMs and MARC‐145 cells. PRRSV‐2 strain FJZHK‐2025 was isolated in PAMs and identified using IFA (Figure 1A). In addition, growth characteristics of the FJZHK‐2025 and the positive control strain FJZ03 were also assessed in PAMs. The results showed that the replication rate of FJZ03 was significantly higher than that of FJZHK‐2025 from 36 to 60 hpi in PAMs (Figure 1B), which further confirmed FJZHK‐2025 was successfully isolated in PAMs. However, the FJZHK‐2025 strain was unable to propagate on MARC‐145 cells (data not shown).

Figure 1FJZHK‐2025 was successfully isolated from PAMs. (A) Result of IFA of PAMs cells. Scale bar = 75 μm. (B) Growth kinetics of FJZHK‐2025 and FJZ03.

**Figure:**
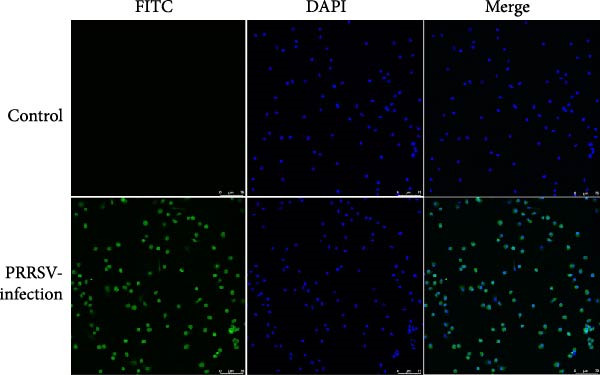
(A)

**Figure:**
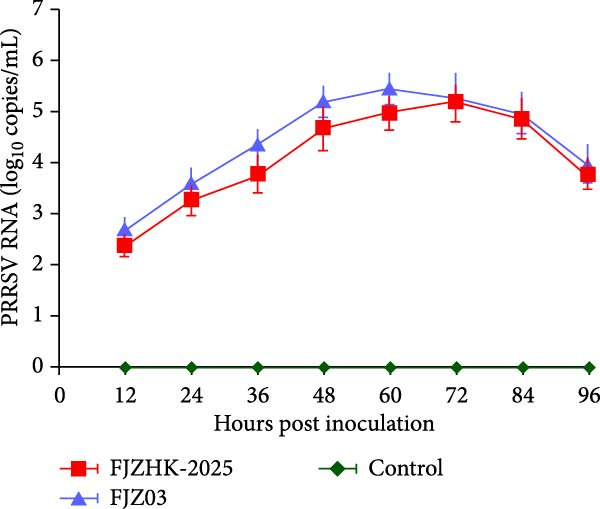
(B)

### 3.3. Genome Analysis

**Figure 2:**
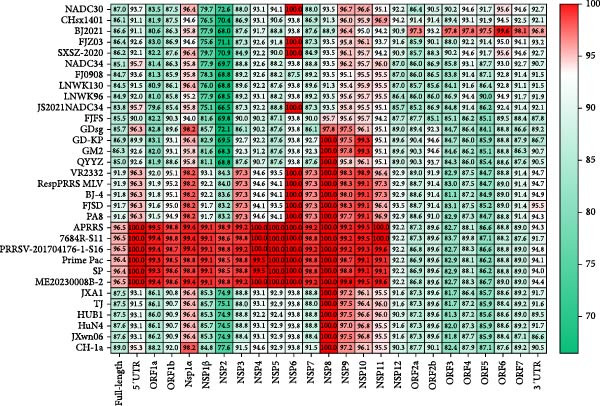
Nucleotide sequence identity (%) of FJZHK‐2025 compared with representative PRRSV‐2 strains.

**Figure 3:**
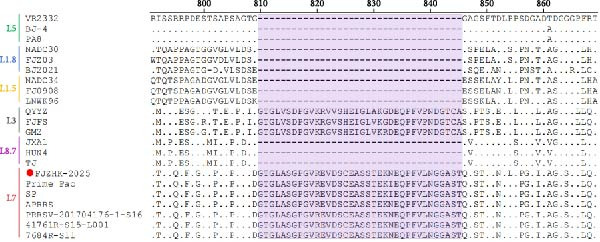
The amino acid alignment of Nsp2 between FJZHK‐2025 and representative PRRSV strains. The 36‐aa insertions (aa810−845) is highlighted in light purple in comparison to VR2332. The strain FJZHK‐2025 in this study is highlighted with a red circle (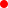).

The genome length of FJZHK‐2025 (GenBank No. PV693702) was 15,520 bp, except for the poly(A) tail. Additionally, genome alignments revealed that FJZHK‐2025 shared 86.4%, 87.0%, 86.6%, 85.1%, 85.0%, 91.9%, 96.4%, 96.5%, and 87.5% identity with FJZ03, NADC30, BJ2021, NADC34, QYYZ, VR2332, Prime Pac, APRRS, and JXA1, respectively. However, it shared only 60.6% identity with LV, suggesting that FJZHK‐2025 belonged to PRRSV‐2 (Supporting Information: Table S1, Figure 2). Comparison of each genomic fragment showed that FJZHK‐2025 had the highest nucleotide identity with Prime Pac‐like strain APRRS in the 5′UTR, ORF1a, and ORF1b regions (Supporting Information: Table S1, Figure 2), while in ORF2‐7 and 3′UTR regions, FJZHK‐2025 had the highest nucleotide identity with the NADC30‐like strain BJ2021 (Supporting Information: Table S1, Figure 2). This finding suggests that FJZHK‐2025 is a recombinant virus. Remarkably, FJZHK‐2025 had 36 consecutive aa insertions (aa810‐845) in the Nsp2 gene compared with VR2332, which are identical to those in Prime Pac‐like strains (L7 PRRSV) and QYYZ‐like strains (L3 PRRSV) (Figure 3). Although these two lineages exhibit similar insertion patterns within the NSP2 gene, their nucleotide and aa homologies are only 76.2%−83.4% and 68.3%−83.2%, respectively. This significant genetic divergence in NSP2 provides a robust molecular basis for distinguishing between the strains of these two lineages.

### 3.4. Phylogenetic and Recombination Analysis

A phylogenetic tree constructed using the complete genome sequences of FJZHK‐2025 and 108 PRRSV reference strains revealed that FJZHK‐2025 belonged to L7 PRRSV‐2 (Prime Pac‐like). In contrast, FJZHK‐2025 belongs to L1.8 PRRSV‐2 based on the ORF5 gene (Figure 4).

Figure 4Phylogenetic tree was constructed based on the full length (A) and ORF5 genes (B) of the FJZHK‐2025 strain and 108 reference PRRSV strains. The reliability of the tree was assessed using a bootstrap analysis with 1000 replications. The FJZHK‐2025 isolate in this study is marked with the black circle (●).

**Figure:**
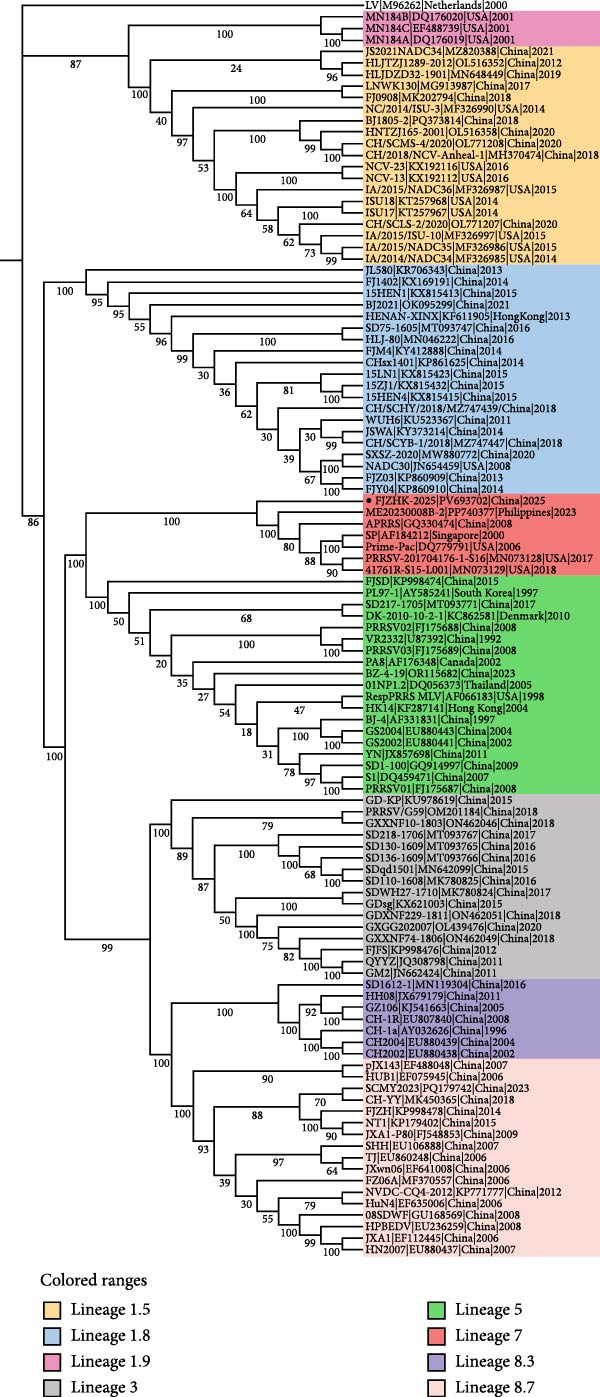
(A)

**Figure:**
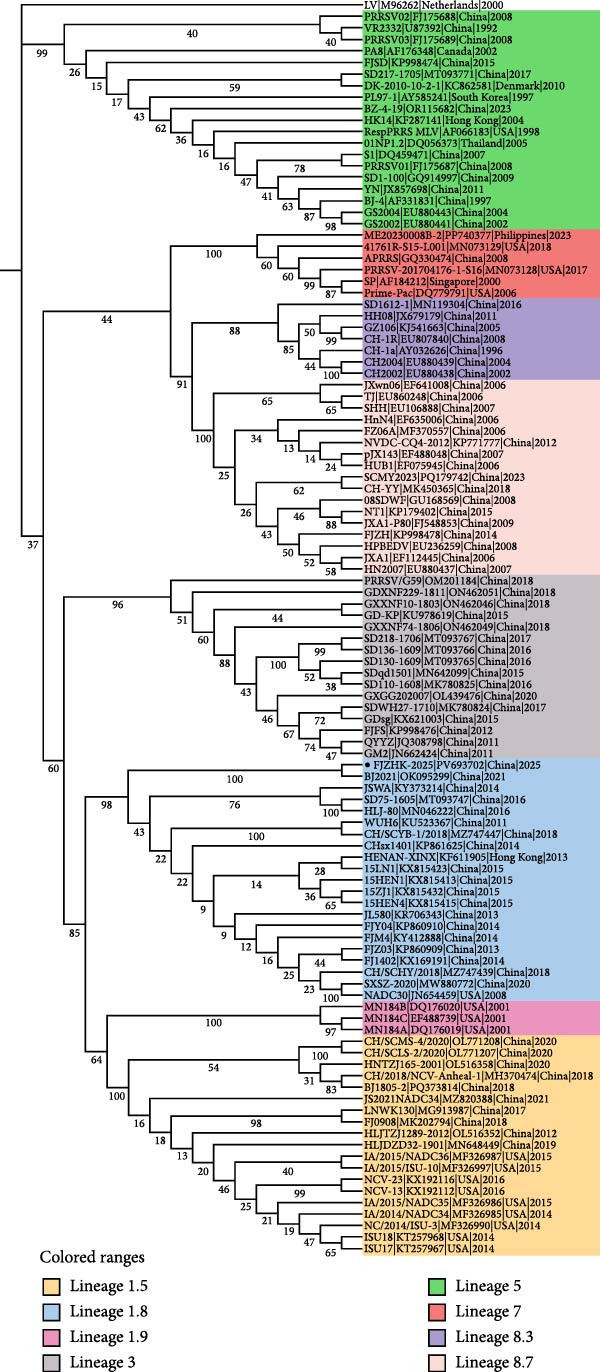
(B)

Potential recombination events within the FJZHK‐2025 genome were analyzed using RDP 4.10 and SimPlot 3.5.1, with the entire genome of FJZHK‐2025 chosen as the query sequence. Prior to recombination analysis, BLAST analysis revealed that the 5^′^UTR‐ORF1b and ORF2a‐3^′^UTR regions of the FJZHK‐2025 sequence exhibited the highest homology with APRRS (99.3%) and BJ2021 (97.8%), respectively. These results suggest that two PRRSV sequences may have contributed partial fragments to FJZHK‐2025 during recombination. RDP 4.10 analysis confirmed that FJZHK‐2025 is a recombinant strain derived from the Prime Pac‐like strain APRRS (L7, the major parent strain) and the NADC30‐like strain BJ2021 (L1.8, the minor parent strain) (Table 1). This recombination event was further verified using SimPlot 3.5.1, and only one recombination breakpoint was identified in the FJZHK‐2025 genome at nt12,236 within the ORF2 region (Figure 5A).

Figure 5(A) Recombination analysis of strain FJZHK‐2025 by Simplot. A similarity plot was generated using the complete genome of FJZHK‐2025 as the query sequence and a sliding window of 200 nt, moving in 20 nt steps. The *y*‐axis shows the percentage similarity between FJZHK‐2025 and parental sequences. Phylogenetic tree analysis of the major parental region (B) and minor parental region (C). The FJZHK‐2025 isolate in this study is marked with the black circle (●).

**Figure:**
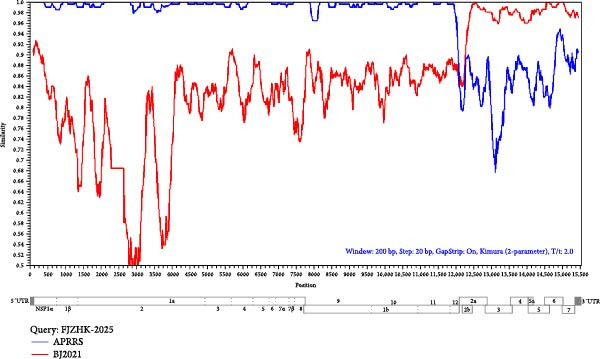
(A)

**Figure:**
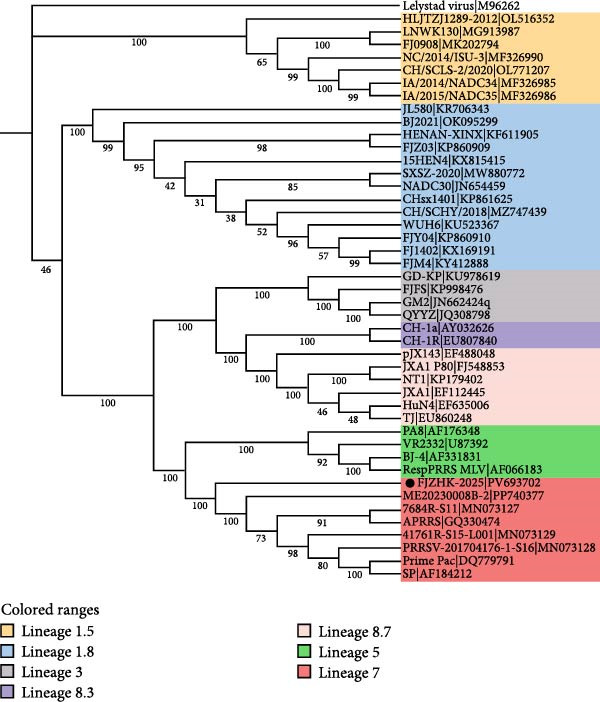
(B)

**Figure:**
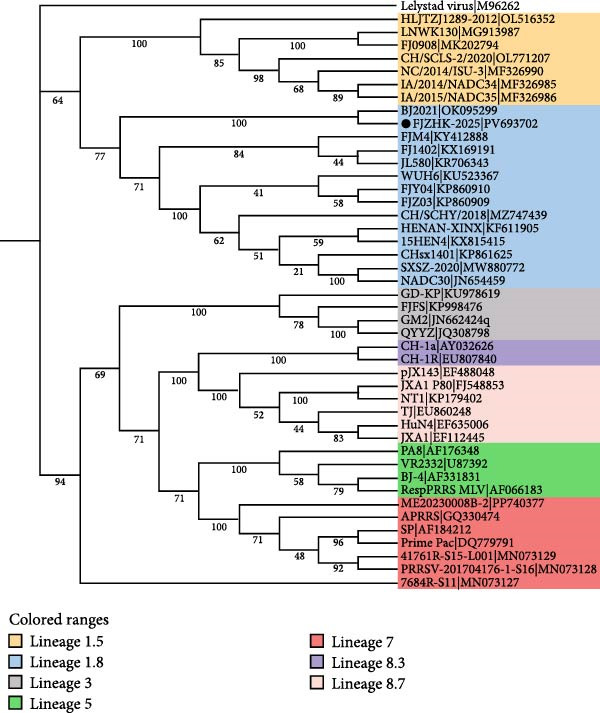
(C)

**Table 1:** Analysis of the FJZHK‐2025 strain recombination event using RDP4.1 software.

Isolate	Breakpoint position in alignment	Major parent	Minor parent	*p*‐Value						
				RDP	GENECONV	Bootscan	MaxChi	Chimera	SiScan	3Seq
FJZHK‐2025	12,336	APRRS	BJ2021	2.845 × 10^−224^	1.091 × 10^−209^	2.038 × 10^−224^	4.104 × 10^−49^	2.945 × 10^−13^	—	1.753 × 10^−10^

Further evidence for recombination was provided by phylogenetic trees constructed based on the two recombinant fragments flanking the potential breakpoint. The results showed that FJZHK‐2025 was closely related to L7 PRRSVs (represented by Prime Pac) in the nt1–12,335 segment (Figure 5B), whereas the remaining fragment (nt12,336–15,520) was closely related to L1.8 PRRSVs (represented by NADC30) circulating in China (Figure 5C).

### 3.5. Clinical Signs of FJZHK‐2025‐Infected Piglets

The group inoculated with FJZHK‐2025 developed a high fever (> 40°C) between 1 and 7 dpi, with the peak temperature reaching 40.4°C at 3 dpi (Figure 6A). Furthermore, the group inoculated with FJZHK‐2025 also exhibited mild to moderate clinical signs, including anorexia, sneezing, coughing, and lethargy from 3 to 9 dpi, with no deaths observed by the end of the experiment. In comparison, the negative control group remained healthy with no clinical symptoms throughout the experiment. Additionally, the ADWG of the FJZHK‐inoculated group was lower than that of the control group, though the difference was not statistically significant (*p* > 0.05) (Figure 6B).

Figure 6The rectal temperature, average daily weight gain, viral loads, and PRRSV‐specific antibody levels of piglets in FJZHK‐2025‐inoculation and control groups. (A) Rectal temperature of piglets in the two groups. (B). Average daily weight gain of piglets in FJZHK‐2025‐inoculation and control groups. (C) Viral load in sera of piglets at different days in each group. (D) Antibody level of PRRSV N protein of piglets at different days in each group.

**Figure:**
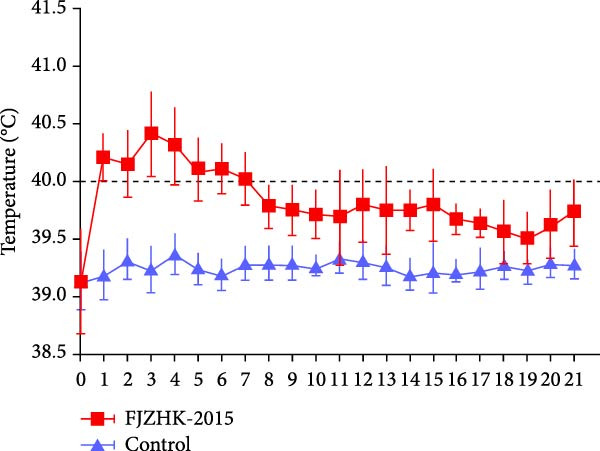
(A)

**Figure:**
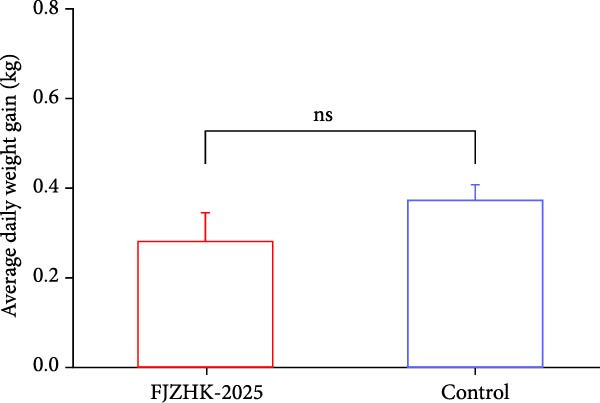
(B)

**Figure:**
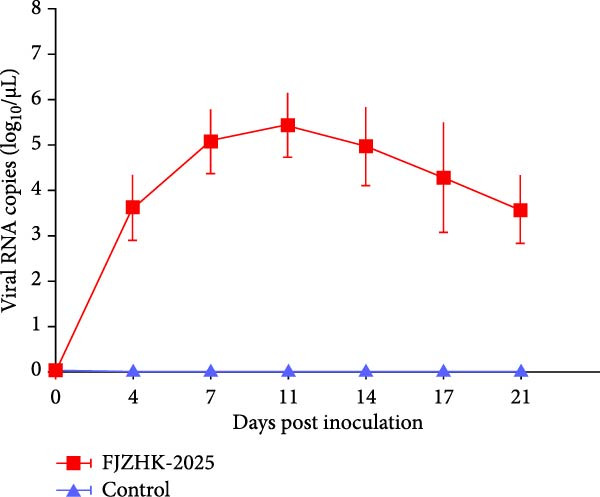
(C)

**Figure:**
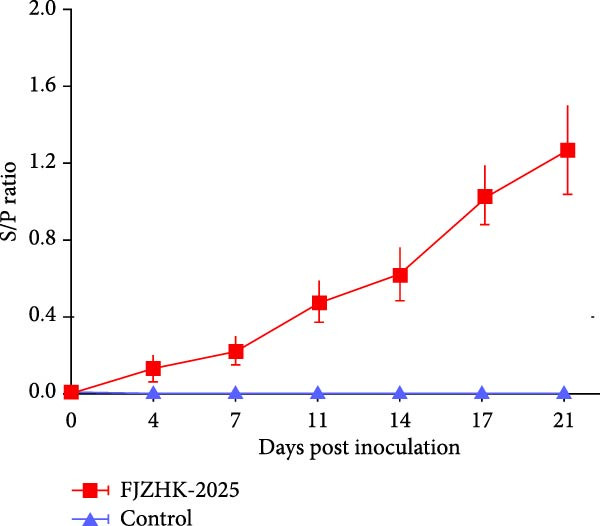
(D)

Serum viral loads in piglets from each group were quantified via RT‐qPCR. As shown in Figure 6C, viremia was detected from 4 to 21 dpi, with peak levels observed at 11 dpi. Levels of serum antibodies specific to the PRRSV N protein were measured using the IDEXX ELISA kit. All piglets (5/5) in the FJZHK‐2025‐inoculated group seroconverted at 11 dpi, with PRRSV antibody levels increasing gradually thereafter (Figure 6D). Notably, neither viral RNA nor PRRSV serum antibodies were detected in the negative control group during the experiment (Figure 6).

At necropsy, lung tissue samples from piglets infected with FJZHK‐2025 displayed diffuse consolidation lesions (Figure 7B), and the mean lung lesion score in FJZHK‐2025‐inoculation group (22.5) was significantly higher than those of the control group (2.5) (*p* < 0.01). Histopathological examination further revealed that all piglets infected with FJZHK‐2025 had severe interstitial pneumonia, characterized by thickened alveolar septa and extensive infiltration of inflammatory cells (Figure 7D). Consistently, the mean microscopic lung lesion score of the FJZHK‐2025‐inoculation group (1.6) was significantly higher than that in the control group (*p* < 0.01). The results of IHC staining revealed that PRRSV‐positive signals were observed in the macrophages and bronchial epithelial cells in the FJZHK‐2025‐inoculation group (Figure 7F). In contrast, no pathological lesions or PRRSV‐positive staining were observed in control piglets (Figure 7A,C,E).

Figure 7Gross and microscopic lesions, and IHC examination of the lungs in the FJZHK‐2025‐inoculation and control groups. Gross pathological lung changes in the FJZHK‐2025‐inoculation group (B) and the control group (A) at 21 dpi. Microscopic pathological lung changes in the FJZHK‐2025‐inoculation group (D) and the control group (C) at 21 dpi. IHC examination results for the lung of the FJZHK‐2025‐inoculation group (F) and the control group (E).

**Figure:**
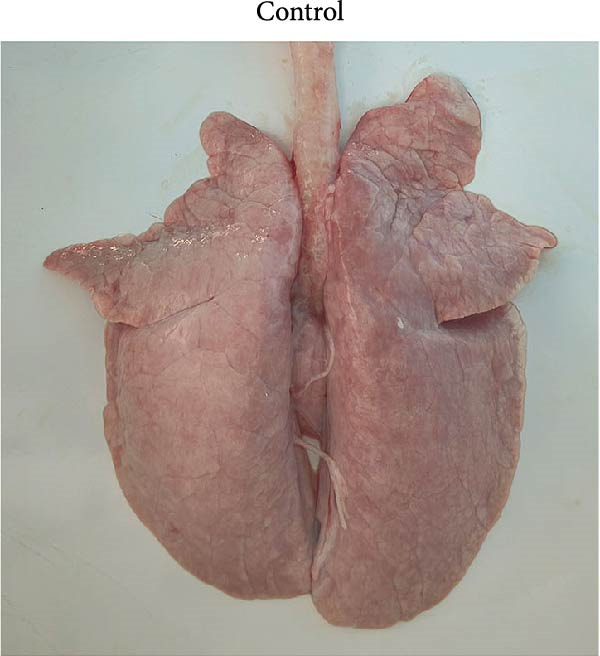
(A)

**Figure:**
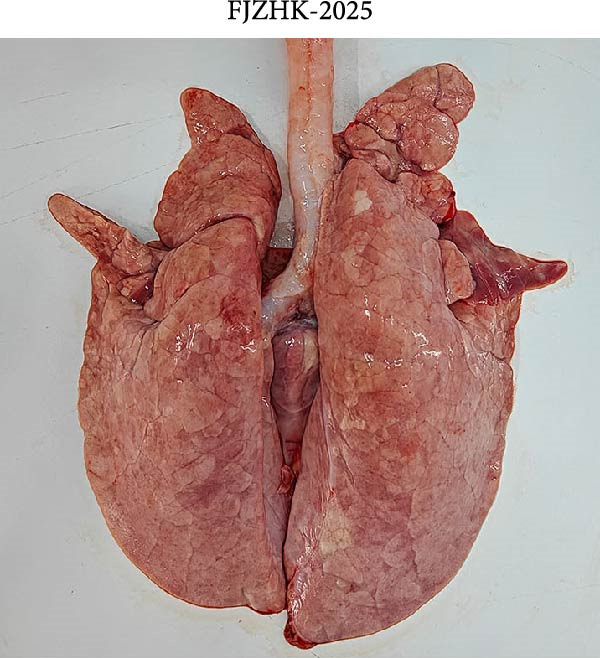
(B)

**Figure:**
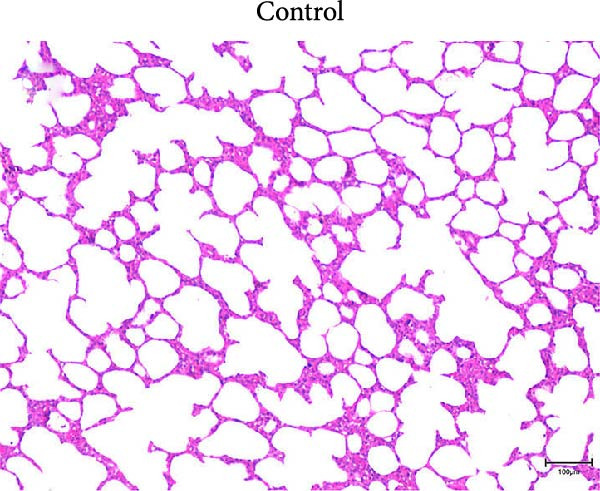
(C)

**Figure:**
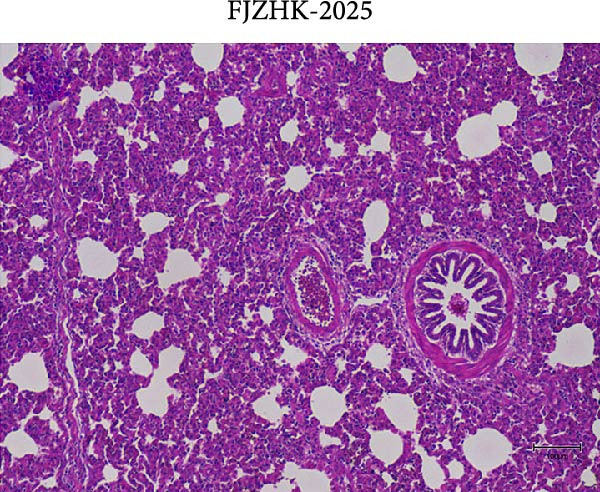
(D)

**Figure:**
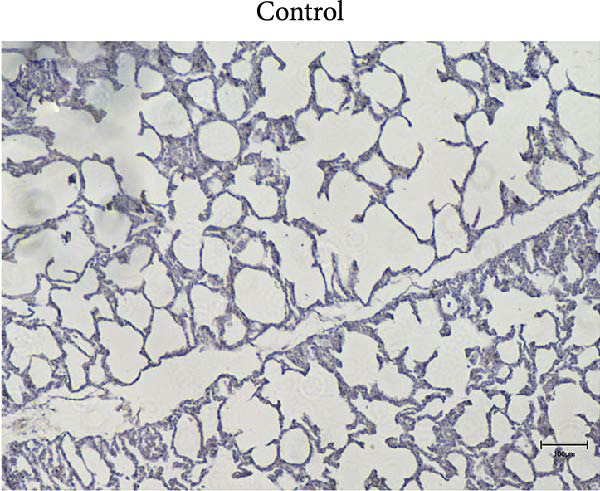
(E)

**Figure:**
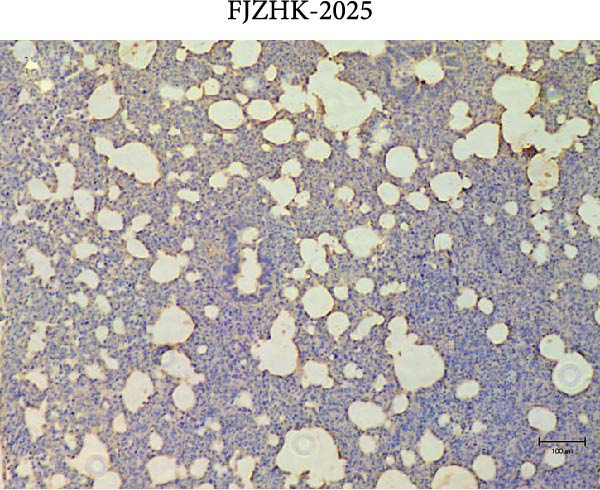
(F)

## 4. Discussion

Six PRRSV‐2 commercial modified‐live virus (MLV) vaccines, including Prime Pac PRRS, Ingelvac PRRS MLV, Prevacent PRRS, Ingelvac PRRS ATP, PRRSGard, and Fostera PRRS, have been used in swine herds in the United States [8, 33]. The Prime Pac PRRS attenuated vaccine (belongs to L7 PRRSV‐2) was developed in 1996, and has been shown to effectively control PRRS‐related reproductive and respiratory diseases [8, 33, 34]. Then it was introduced to the market in 2014 and is currently mainly used in pig farms in Asia, Europe and North America [33]. However, it is not used commercially in China [33]. Furthermore, research has revealed that the sequences identified in L7 PRRSV‐2 in the USA are predominantly vaccine‐like [8]. Currently, four different PRRSV strains (L1, L3, L5 and L8) coexist in Chinese swine herds, with NADC30‐like PRRSV (L1.8) becoming the predominant strain. NADC30‐like PRRSV is characterized by its extensive recombination with field/vaccine strains, which further complicates the prevention and control of PRRS [12, 35]. This study identified a novel PRRSV‐2 strain originating from the recombination of L7 and L1.8 PRRSV strains during an epidemiological survey of PRRSV in China. To our knowledge, this is the first report of this recombination pattern of PRRSV.

BLAST analysis revealed that FJZHK‐2025 belongs to L7 PRRSV‐2 and shared the same 36‐aa insertions pattern in Nsp2 region as that of the Prime Pac‐like PRRSV strains. Furthermore, strain FJZHK‐2025 had the highest nucleotide similarity to the Prime Pac‐like strain APRRS (L7) in the 5′UTR‐ORF1b region and to the NADC30‐like strain BJ2021 (L1.8) in the ORF2‐3′UTR region. Further sequence comparisons of each fragment of the PRRSV genome, recombination analysis, and statistically incongruent phylogenetic trees indicated that FJZHK‐2025 was a recombinant strain derived from between L7 PRRSV‐2 and L1.8 PRRSV‐2. Although recombination breakpoints occur randomly, statistical analyses have revealed that the high‐frequency PRRSV‐2 recombination hotspots in China are concentrated within the NSP9 region and the GP2‐GP3 region [36, 37]. Notably, the GP2 protein plays a key role in viral invasion and replication and induce the host immune system to produce specific neutralizing antibodies [38]. In the present study, breakpoint of the recombinant strain FJZHK‐2025 was located in ORF2, which may endow the recombinant virus with enhanced replicative capacity, altered cellular tropism, and greater immune evasion capabilities [38]. These adaptive changes would enable the virus to persist and transmit more efficiently within pig populations, potentially triggering large‐scale epidemics.

Recombinant strains of PRRSV between L7 and L1/L5 have been previously reported in the USA [37]. This study focuses on the novel PRRSV strain FJZHK‐2025, which originated from a natural recombination event between L7 and L1.8 strains. L7 PRRSV strains have mainly been reported in the USA. Notably, the Prime Pac‐like strain APRRS was first reported in China in 2008 [20] and exhibits 99.8% nucleotide identity with the Prime Pac PRRS vaccine. However, the origin of this strain remains unclear. Currently, only the APRRSV strain of L7 from China is recorded in the GenBank database, suggesting that L7 PRRSV‐2 may be restricted to specific environments, such as laboratories, rather than being endemic in China. Considering that China has regularly traded animals or animal products with the USA and other countries in recent years, it is possible to speculate that Chinese L7 PRRSV‐2 strains may have originated in the US and then were introduced to China. Nevertheless, more evidence, such as genetic tracing across regions and historical surveillance data, is needed to confirm this hypothesis.

Studies have shown that recombination occurs between field PRRSV/MLV strains and some MLV strains (e.g., HP‐PRRS MLV and Ingelvac PRRS MLV), resulting in the generation of virulent strains with higher virulence than vaccine strains (e.g., FJXS15, SCN17 and TJnh150) (Supporting Information: Table S2) [39–52]. Pigs vaccinated with the Prime Pac PRRS vaccine did not exhibit any adverse reactions or hyperthermia under both laboratory and field conditions [53, 54]. Based on cumulative data, NADC30‐like PRRSVs exhibit moderate to high pathogenicity in piglets in inoculation study [41, 55–58]. Viremia is one of the measures of PRRSV virulence [59]. In the present study, viremia was detected in all challenge groups using RT‐qPCR, and all piglets infected with FJZHK were viremic from 4 dpi to 21 dpi. The virulence of PRRSV and the duration of the fever it induces are generally positively correlated with its pathogenic traits [60, 61]. In this study, animals infected with FJZHK‐2025 exhibited persistent high fevers (>40.0°C from 1 to 7 dpi), mild to moderate clinical signs and moderate lung lesions, suggesting that FJZHK‐2025 could be considered a mildly to moderately virulent strain. The results of this study further suggest that recombination may play an important role in the evolution and virulence of PRRSV. However, the lack of comparative pathogenicity studies involving Prime Pac‐like or NADC30‐like strains, coupled with the small sample size, may affect our assessment of the pathogenicity and transmissibility of recombinant strains in pigs.

To date, NADC30‐like PRRSV has undergone complex recombination with four PRRSV‐2 lineages (L1.8, L3, L5 and L8), which has further increased PRRSV evolution [62]. The emergence of the L7 and L1.8 recombinant strains will make clinical virus detection more difficult, increase the risk of live vaccines and potentially cause new outbreaks. Currently, the live attenuated PRRSV‐2 vaccine is one of the most important means for controlling and preventing PRRS in China. Although eight commercial MLV vaccines (Ingelvac PRRS MLV/RespPRRS MLV, CH‐1R, HuN4‐F112, JXA1‐P80, R98, TJM‐F92, GDr180, and PC) are used to control and prevent PRRSV in China, these MLV vaccines offer only partial cross‐protection against heterologous NADC30‐like strains [63]. Therefore, the correct choice of vaccine is essential for the prevention and control of L7 PRRSV. In general, vaccines tend to offer better protection against more similar strains [64]. Sequence alignment revealed that FJZHK‐2025 shared the highest homology with the Ingelvac PRRS MLV (91.8%). Therefore, the Ingelvac PRRS MLV vaccine could potentially be used to prevent and control L7 PRRSV in swine herds, although this requires further validation. Furthermore, when designing the next generation of PRRS vaccines, efforts should focus on enhancing their cross‐protection capabilities. A possible approach would be to design PRRSV consensus sequences or anti‐recombinant vaccine to achieve high‐efficiency heterologous protection [62, 65]. In addition to the aforementioned aspects, sensitive and specific diagnostic assays are important tools for controlling PRRSV. Notably, the unique genetic features of the Nsp2 gene in L7 PRRSV‐2 pose a significant challenge to existing diagnostic methods in China. These established diagnostic approaches were specifically developed based on the Nsp2 gene sequences from different PRRSV‐2 lineages (e.g., C‐PRRSV, HP‐PRRSV and NADC30‐like PRRSV) to enable the rapid detection of PRRSV in cases of mixed infection and precise differentiation of different PRRSV lineages [66–70]. However, due to the significant genetic divergence of the Nsp2 gene in L7 PRRSV‐2 from those of the aforementioned lineages, these diagnostic methods may be unable to detect L7 PRRSV‐2. Two commercially available RT‐qPCR detection kits targeting the PRRSV Nsp2 gene were selected to evaluate the efficacy of commercial diagnostic tools in identifying L7 PRRSV strains. RT‐qPCR detection kits yielded negative results for L7 PRRSV strains, with no specific fluorescent signal amplification observed (data not shown), confirming that current commercial kits lack the sensitivity and specificity required for the accurate detection of the emerging L7 PRRSV strain. Furthermore, the emergence of this new virus highlights the critical importance of strict biosecurity measures for preventing and controlling PRRS [27]. Pig farms should thus implement the following measures: (1) upgrade biosecurity systems and adopt all‐in/all‐out production patterns to prevent the spread of these recombinant viruses across major pig‐producing provinces, (2) strengthen monitoring and regulation of imported live pigs and vaccine‐related materials to avoid introducing high‐risk viral strains, (3) establish a comprehensive surveillance network to enable real‐time risk assessment and rapid response to potential PRRSV outbreaks. In addition to the aforementioned biosecurity strategies, pig farms should implement the following targeted measures to reduce the risk of PRRSV recombination: (1) use a single PRRSV MLV within the same swine herd, (2) conduct routine testing on introduced gilts and minimize the mixing of piglets infected with different PRRSV strains, (3) conduct genome‐wide or multigene monitoring of PRRSV isolates to enable the timely detection of new strains.

In conclusion, the novel PRRSV‐2 strain FJZHK‐2025 arose from recombination of Lineage 7 and Lineage 1.8 PRRSV‐2 strains. FJZHK‐2025 exhibits mild to moderate virulence in piglets. These findings provide valuable evidence to help us understand the role of genomic recombination in PRRSV evolution.

## Conflicts of Interest

The authors declare no conflicts of interest.

## Author Contributions

Yuan Yang and Haoyu Chen contributed equally to this work.

## Funding

This study was supported by Leading Project Foundation of Science Department of Fujian Province (Grants 2021N0032 and 2025N0012), Natural Science Foundation of Fujian Province (Grant 2023J01989), Doctoral Initiation Grant (Grant LB2021009), Innovative Star Talent Program of Fujian Province ([2023]110) and Research Foundation of Fujian Provincial Key Laboratory for Prevention and Control of Animal Infectious Diseases and Biotechnology (Grant ZDSYS2023005).

## Supporting Information

Additional supporting information can be found online in the Supporting Information section.

## Supporting information


**Supporting Information** Table S1. Nucleotide and amino acid sequence identity (%) of FJZHK‐2025 as compared to representative PRRSV strains. The table provides detailed information on the nucleotide and amino acid sequence identities (%) between FJZHK‐2025 and 32 different lineages (1.5, 1.8, 3, 5, 7 and 8) of PRRSV‐2, as well as one PRRSV‐1 strain (LV). Table S2. Potential recombination events between MLV and field/MLV strains of PRRSV‐2. The table contains a brief description of recombinant strains, GenBank accession numbers, isolation region, recombination cross‐over region, parental viruses, and pathogenicity/clinical manifestations.

## Data Availability

The data used to support the findings of this study are included within the article and at GenBank database.
