# Supplementary material for: Genetic and Pathogenic Characteristics of an Emerging Lineage 7 PRRSV‐2 (Prime Pac‐Like) Strain in China
Source: Transbound Emerg Dis. 2025 Dec 18;2025:8104154. doi: 10.1155/tbed/8104154 (PMC12715340; doi:10.1155/tbed/8104154)
Supplement: Supplementary file 1 — Supporting Information Table S1. Nucleotide and amino acid sequence identity (%) of FJZHK‐2025 as compared to representative PRRSV strains. The table provides detailed information on the nucleotide and amino acid sequence identities (%) between FJZHK‐2025 and 32 different lineages (1.5, 1.8, 3, 5, 7 and 8) of PRRSV‐2, as well as one PRRSV‐1 strain (LV). Table S2. Potential recombination events between MLV and field/MLV strains of PRRSV‐2. The table contains a brief description of recombinant strains, GenBank accession numbers, isolation region, recombination cross‐over region, parental viruses, and pathogenicity/clinical manifestations. [file TBED-2025-8104154-s001.pdf]

Table S1. Nucleotide and amino acid sequence identity (%) of FJZHK-2025 as compared to representative PRRSV strains

Regions	NADC30	CHsx1401	BJ2021	FJZ03	SXSZ-2020	IA/2014/NADC34	FJ0908	LNWK130	LNWK96	JS2021NADC34	FJFS	GDsg	GD-KP	GM2	QYYZ	VR2332	RespPRRS MLV	BJ-4	FJSD	PA8	APRRS	7684R-S11	PRRSV-201704176-1-S16	Prime Pac	SP	ME20230008B-2	JXA1	TJ	HUB1	HuN4	JXwn06	CH-1a	LV
	lineage 1.8				lineage 1.5						lineage 3					lineage 5					lineage 7						lineage 8						PRRSV-1
	PRRSV-2																																
Full-length	87.0/	86.1/	86.6/	86.4/	86.2/	85.1/	84.7/	84.5/	84.9/	83.8/	85.5/	85.7/	86.9/	86.3/	85.0/	91.9/	91.9/	91.8/	91.7/	91.6/	96.5/	96.5/	96.5/	96.4/	96.4/	96.5/	87.5/	87.5/	87.5/	87.6/	87.6/	89.0/	60.6/
5'UTR	93.7/	91.1/	91.1/	92.6/	92.1/	95.7/	93.6/	91.5/	92.0/	95.7/	90.0/	96.3/	89.9/	92.6/	92.6/	96.3/	96.3/	96.3/	96.3/	96.3/	100/	100/	100/	100/	100/	100/	93.1/	91.5/	93.1/	93.1/	93.1/	95.3/	61.9/
ORF1a	83.5/	81.9/	80.6/	83.0/	82.2/	81.4/	81.3/	80.9/	81.0/	79.6/	82.2/	82.8/	83.1/	82.0/	81.9/	92.0/	91.9/	91.8/	91.6/	91.5/	99.5/	99.4/	99.4/	99.3/	99.3/	99.4/	86.1/	86.1/	86.0/	86.1/	86.2/	88.2/	55.9/
ORF1b	87.6/	87.7	86.5/	87.0/	87.4/	86.6/	86.2/	86.4/	86.1/	86.0/	90.3/	89.6/	93.1/	93.1/	88.6/	95.1/	95.2/	95.1/	95.0/	94.9/	98.8/	98.8/	98.7/	98.5/	98.6/	98.6/	90.8/	90.7/	90.9/	90.7/	90.9/	92.0/	63.1/
ORF2-7	91.4/	90.8/	97.9/	90.4/	91.2/	88.8/	87.9/	87.7/	89.8/	87.6/	86.4/	86.4/	87.1/	86.7/	86.8/	86.8/	86.8/	86.7/	86.9/	86.7/	86.4/	86.7/	86.3/	86.2/	86.2/	86.4/	85.8/	85.9/	85.8/	86.0/	85.8/	86.4/	66.7/
Nsp1α	90.6/96.4	87.8/94.6	87.6/95.8	88.6/94.6	90.2/96.4	86.9/95.8	87.1/95.8	88.2/96.4	87.8/95.2	86.1/95.8	89.0/94.0	93.8/98.2	88.6/96.4	90.2/95.8	90.8/95.8	96.0/98.2	96.0/98.2	95.8/98.2	96.0/98.2	95.8/98.2	99.6/99.4	99.6/99.4	99.4/99.4	99.2/98.8	99.2/98.8	99.4/99.4	92.0/96.4	92.4/96.4	92.4/96.4	92.0/96.4	92.4/96.4	94.8/98.2	63.4/66.9
NSP1β	84.3/79.7	81.3/76.5	80.6/77.9	82.2/75.6	84.3/79.7	80.5/77.9	80.3/78.3	79.0/76.0	80.8/77.9	79.0/75.1	83.1/81.6	86.9/85.7	84.8/82.9	82.5/81.6	82.9/81.1	94.9/93.1	94.6/92.2	94.8/92.2	94.5/91.7	94.2/91.7	99.4/99.1	99.4/99.1	99.2/99.1	99.2/99.1	99.2/99.1	99.2/99.1	86.8/85.3	87.1/85.7	86.3/85.3	87.1/85.7	87.1/85.3	88.3/84.8	50.1/44.1
NSP2	79.3/72.6	77.7/70.4	75.5/68.0	78.4/71.1	78.5/70.9	77.8/69.7	77.4/68.8	77.2/68.8	77.0/68.5	75.2/66.5	76.9/69.8	78.1/72.1	76.7/69.5	76.2/68.3	76.4/68.8	88.9/84.3	88.8/84.0	88.7/83.6	88.3/83.2	88.1/83.2	99.3/98.9	99.1/98.6	99.2/98.8	99.1/98.5	99.1/98.5	99.2/98.8	81.0/74.9	81.0/75.1	80.8/74.9	80.9/74.5	81.1/74.9	83.2/77.6	51.8/31.7
NSP3	86.5/91.5	85.7/89.9	84.9/91.5	87.6/91.3	85.4/90.6	85.8/91.5	85.6/90.8	85.0/90.4	84.3/89.9	84.8/90.8	85.1/90.6	83.4/90.1	86.8/92.2	85.0/90.6	85.1/90.6	94.2/96.4	94.3/96.6	94.2/96.4	93.9/96.4	94.0/96.6	99.6/100	99.4/100	99.6/100	99.3/99.8	99.3/99.8	99.5/99.8	90.1/94.2	90.1/94.4	90.2/94.4	90.2/94.2	90.1/94.6	92.5/95.3	59.3/58.2
NSP4	84.3/93.1	83.5/93.1	84.0/91.7	85.8/92.6	83.7/92.2	84.2/92.6	84.2/92.6	83.0/92.2	84.5/93.1	82.5/92.2	84.8/90.2	84.8/90.2	87.9/92.2	85.0/91.2	84.8/90.7	91.0/94.6	91.0/94.6	91.0/94.6	90.7/94.1	90.8/94.6	99.5/100	99.5/100	99.5/100	99.3/99.5	99.3/99.5	99.5/100	88.2/93.1	88.4/93.1	87.7/92.2	88.2/93.1	88.4/93.1	90.7/94.6	60.4/62.6
NSP5	91.4/94.1	88.4/90.6	85.7/88.8	90.0/91.8	89.0/90.0	85.1/88.2	85.3/88.2	85.3/87.6	84.3/88.2	83.1/88.8	83.9/87.1	83.7/87.6	83.5/87.6	83.7/87.6	83.7/87.6	92.9/93.5	92.9/94.1	92.9/94.1	92.9/93.5	92.5/94.1	100/100	100/100	100/100	100/100	100/100	100/100	89.0/92.9	89.4/92.9	89.2/92.4	89.6/92.9	89.6/92.9	92.0/92.9	64.4/70.6
NSP6	91.7/100	89.6/93.8	87.5/93.8	91.7/100	89.6/100	85.4/93.8	83.3/87.5	85.4/93.8	85.4/93.8	87.5/100	91.7/93.8	91.7/93.8	91.7/93.8	91.7/93.8	91.7/93.8	93.8/100	93.8/100	93.8/100	93.8/100	93.8/100	97.9/100	97.9/100	97.9/100	97.9/100	97.9/100	97.9/100	93.8/93.8	93.8/93.8	93.8/93.8	93.8/93.8	93.8/93.8	91.7/93.8	66.7/81.2
NSP7	85.5/88.0	84.7/86.9	82.6/87.6	84.6/87.3	81.1/84.9	82.5/88.8	83.1/89.2	82.6/89.6	82.6/89.2	81.0/87.3	86.7/90.0	84.2/86.1	88.0/92.7	87.9/92.3	85.6/87.6	93.8/97.3	93.8/97.3	93.7/97.3	93.6/97.3	94.0/97.3	99.7/99.2	99.6/98.8	99.7/99.2	99.6/98.8	99.6/98.8	99.6/99.2	87.5/88.8	87.5/88.0	87.8/88.8	87.8/88.8	87.6/88.4	89.8/91.5	53.4/48.8
NSP8	91.3/93.5	91.3/91.3	87.5/88.9	90.6/93.5	86.2/93.5	94.2/93.5	92.8/93.5	90.6/91.3	91.3/93.5	92.0/93.5	91.3/95.7	93.5/97.8	96.4/100	95.7/100	92.0/100	96.4/100	96.4/100	96.4/100	97.1/100	95.7/100	100/100	100/100	100/100	100/100	100/100	100/100	94.9/100	95.7/100	94.9/100	94.9/100	95.7/100	95.7/100	65.2/69.6
NSP9	87.0/96.7	86.7/96.0	85.9/96.4	86.4/95.8	87.3/96.1	86.7/96.2	86.0/95.1	86.3/95.4	85.9/95.6	86.7/95.8	91.9/95.6	92.0/97.5	94.2/97.5	94.3/97.8	89.7/95.8	94.5/98.3	94.5/98.3	94.3/98.0	94.4/98.3	94.2/97.7	99.2/99.2	99.2/99.2	99.2/99.2	98.9/98.7	99.0/98.8	99.3/99.8	92.1/97.2	92.0/97.5	92.2/97.5	92.0/97.0	92.2/97.5	93/97.2	66.4/74.5
NSP10	87.2/96.6	85.9/95.9	86.1/95.0	86.6/96.1	87.2/95.7	86.9/95.7	86.8/95.5	87.0/95.9	86.5/95.7	86.5/95.5	91.4/95.7	89.1/96.1	96.8/99.3	96.8/99.3	88.6/96.1	97.1/98.9	97.1/99.1	97.1/99.1	96.7/99.1	96.9/99.1	99.5/99.5	99.5/99.5	99.3/99.3	99.2/99.1	99.2/99.1	99.3/99.5	90.4/96.1	90.5/96.4	90.6/96.6	90.5/96.8	90.6/96.8	92.6/96.1	61.2/65.1
NSP11	90.3/95.1	93.9/96.9	87.9/94.2	89.2/93.7	87.7/94.2	87.0/96.0	86.7/95.5	86.8/95.5	87.0/95.5	85.2/95.1	88.0/94.2	86.7/95.1	88.8/95.1	88.9/95.1	88.3/95.1	95.5/96.4	96.0/97.3	95.8/97.3	95.5/96.4	95.7/96.9	99.9/100	100/100	99.7/99.6	99.6/99.1	99.6/99.1	99.6/99.6	90.9/95.5	90.9/96.0	90.7/96.0	90.7/95.5	90.9/95.5	92.8/95.5	66.1/75.8
NSP12	87.7/92.2	87.9/94.2	87.7/90.9	87.7/91.6	87.9/90.9	84.8/87.0	84.4/87.0	84.4/87.0	84.4/86.4	82.9/85.7	83.5/87.7	84.6/89.0	84.0/89.6	83.5/89.6	84.0/89.0	91.6/92.9	91.8/92.9	92.0/92.9	92.0/93.5	91.3/92.9	93.5/92.2	93.5/92.2	93.5/92.2	93.5/92.2	93.5/92.2	93.5/92.2	86.1/91.6	86.1/91.6	86.1/90.9	85.9/91.6	86.1/91.6	84.8/90.3	50.0/42.0
ORF2	86.4/88.3	87.3/90.3	97.3/94.2	85.9/87.2	85.7/86.0	86.0/83.3	86.0/82.5	85.7/82.1	86.0/82.9	85.2/82.1	87.7/85.2	89.4/89.1	90.4/90.3	90.3/89.9	90.3/90.3	89.0/87.2	88.7/86.8	88.6/86.4	88.7/86.4	88.6/86.4	87.2/85.2	87.3/85.6	86.8/84.0	86.9/84.4	86.9/84.4	86.8/84.4	87.3/84.8	87.3/84.8	87.2/84.8	87.3/84.4	87.3/84.8	87.7/85.6	66.9/65.2
ORF3	90.2/89.8	89.4/89.0	97.8/96.9	88.0/86.3	90.2/89.8	85.8/85.1	83.8/81.6	84.1/82.4	86.9/87.1	84.8/83.9	85.1/85.5	84.7/85.1	84.7/83.9	84.6/83.9	84.3/84.7	82.9/81.6	83.0/82.4	81.1/82.5	83.1/82.7	82.9/82.4	82.7/81.2	83.7/82.4	82.7/81.2	82.9/81.6	82.9/81.6	82.7/81.2	81.7/80.0	81.7/80.4	81.3/79.6	82.0/80.4	81.8/80.4	82.4/81.2	65.5/57.9
ORF4	94.6/93.3	93.1/93.3	97.8/95.5	92.2/92.2	94.6/93.3	93.1/93.9	91.4/90.5	91.6/91.1	94.4/93.9	91.4/94.4	86.2/88.8	86.4/87.7	86.0/88.3	86.2/88.3	86.0/88.8	87.5/87.2	87.5/87.2	87.2/86.0	87.5/87.2	87.3/86.0	88.1/88.3	89.8/88.3	88.3/88.3	88.1/87.7	88.1/87.7	88.3/88.3	86.4/88.8	87.2/89.9	87.2/89.9	87.3/90.5	87.7/90.5	87.9/89.4	68.9/70.4
ORF5	91.7/90.5	91.9/91.5	97.5/95.5	91.4/90.0	91.7/90.5	87.7/89.6	87.1/89.6	86.4/88.1	90.0/93.0	86.2/88.1	85.1/84.1	84.1/82.6	85.9/83.6	85.1/83.6	85.4/84.1	84.7/80.6	84.7/81.1	84.9/81.1	85.2/81.6	84.7/80.6	86.6/83.6	87.1/85.6	86.6/83.6	86.2/83.1	86.2/83.1	86.7/83.6	85.7/81.6	85.9/82.1	85.6/82.1	85.9/82.1	85.9/82.1	87.1/82.6	63.6/56.3
ORF6	95.6/97.1	94.5/97.1	99.6/98.9	95.0/96.6	95.6/97.1	93.0/94.3	92.8/95.4	92.8/95.4	94.9/97.1	92.4/94.9	89.5/92.6	88.8/93.7	88.8/93.7	88.8/92.0	88.6/92.0	88.8/93.1	89.0/93.1	89.0/93.1	89.0/93.7	88.8/93.1	88.8/94.3	88.2/94.3	88.8/94.3	88.8/94.3	88.8/94.3	89.0/94.9	88.6/93.7	88.4/93.7	88.6/93.7	88.6/93.7	88.4/93.7	87.6/92.6	70.9/81.0
ORF7	94.6/96.8	92.5/94.4	98.1/99.2	94.1/96.0	94.6/96.8	92.7/96.0	91.4/94.4	91.1/93.5	91.7/92.7	91.4/92.7	88.4/88.7	86.6/90.3	87.9/88.7	86.3/87.9	87.6/90.3	91.4/91.1	91.4/91.1	91.4/91.1	91.4/91.1	91.4/91.1	89.0/91.1	87.6/89.5	89.0/91.1	89.0/91.1	89.0/91.1	89.0/91.1	89.2/89.5	89.2/89.5	89.0/89.5	89.2/89.5	87.1/85.5	89.2/89.5	66.9/60.3
3'UTR	92.7/	92.1/	96.8/	93.2/	92.7/	90.7/	91.5/	91.5/	91.9/	92.1/	87.8/	89.2/	86.7/	90.7/	90.5/	94.7/	94.7/	94.9/	95.5/	94.3/	94.3/	91.7/	94.8/	94.1/	94.0/	94.4/	91.7/	91.6/	89.5/	91.7/	86.6/	90.5/	73.3/

Table S2. Potential recombination events between MLV and field/MLV strains of PRRSV-2

No.	Recombinant virus	Accession number	Isolation region	Cross-over region	Parental virus		pathogenicity or Clinical manifestation	Reference
					Major	Minor		
1	FJZHK-2025	PV693702	China	ORF2	MLV-like Strain APRRS	BJ2021	mild to moderate virulence in piglets, with high fevers, moderate clinical signs, and lung lesions	This study
2	Em2007	EU262603	China	Nsp2; Nsp9	CH-1R/vaccine	WUH1/HP-PRRSV	Em2007 is a highly pathogenic PRRSV strain with a virulence level higher than that of the CH-1a parental strain of the CH-1R strain but lower than that of the HP-PRRSV WUH1 strain	[39]
3	GDsg	KX621003	China	5′UTR-NSP2; NSP7- NSP10; ORF3	QYYZ	JXA1-P80/Vaccine	higher virulence in pigs than QYYZ and JXA1-P80	[40]
4	FJXS15	KX758250	China	Nsp1-ORF4	JXA1 P80/Vaccine	FJZ03	a highly virulent strain of PRRSV resulted in a mortality rate of 25% in piglets.	[41]
5	TJnh1501	KX510269	China	Nsp2	NADC30	MLV-like strain TJbd14-1	intermediate virulence for piglets, lower pathogenicity than HP-PRRSV JXwn06, but higher than NADC30-like CHsx1401 for piglets	[42]
6	GM2	JN662424	China	ORF1b	QYYZ	RespPRRS MLV/Vaccine	persistent higher fever, obvious clinical performance and lung lesions	[43]
7	SCN17	MH078490	China	5′-UTR- nsp1β; nsp3-nsp7; nsp9-nsp11	NADC30	RespPRRS MLV/Vaccine	a moderately virulent strain with lower pathogenicity than that of the HP-PRRSV strain SCwhn09CD.	[44]
				ORF5	NADC30	JXA1		
8	GX2024	PV362838	China	5′-UTR-ORF1a; ORF1a-ORF1b	JXA1	NADC30	a highly pathogenic PRRSV strain with high fever, high morbidity and mortality for piglets	[45]
				ORF3-ORF4	RespPRRS MLV/Vaccine	NADC30		
9	HeN1301	MF766470	China	Nsp2	MLV-like strain TJbd14-1	HeNan-A2	unknown	[46]
	HeN1401	MF766471	China	Nsp2; ORF2	NADC30	MLV-like strain TJbd14-1		
	HeN1601	MF766474	China	Nsp2; ORF2-ORF6	NADC30	MLV-like strain TJbd14-1		
10	PRRSV2/swine/Indiana/100837/2023	PV607082	USA	Nsp9; ORF5	Prevacent PRRS/Vaccine	Fostera PRRS/Vaccine	unknown	[47]
11	USA/IN105404/2021	OQ145436	USA	ORF1b	RespPRRS MLV/Vaccine	Prevacent PRRS/Vaccine	unknown	[48]
12	IA70388-R	MK796165	USA	ORF1a	Fostera PRRS/Vaccine	IA76950-WT	unknown	[49]
13	KU-N1202/2012	MK057529	Korea	NSP11	K07-2273/2007	RespPRRS_MLV/Vaccine	mild-to-moderate clinical signs and mild histopathological changes	[50]
14	GGYC45/2010	PE017913	Korea	NSP5-NSP8	K07-2273/2007	RespPRRS_MLV/Vaccine	unknown	[50]
	GBGJ22/2011	PE017915	Korea	NSP8-NSP12	K07-2273/2007	RespPRRS_MLV/Vaccine		
15	JBNU-22-N01	OP970983	Korea	Nsp4-Nsp7α	NADC34	RespPRRS MLV/Vaccine	unknown	[51]
16	20D160-1	OM681585	Korea	ORF2-ORF4	MLV-like Strain P129	KNU-1902	unknown	[52]
17	21R2-63-1	OM681586	Korea	ORF1b	NADC30	MLV-like Strain P129	unknown	[52]
